# Ropivacaine-Induced Contraction Is Attenuated by Both Endothelial Nitric Oxide and Voltage-Dependent Potassium Channels in Isolated Rat Aortae

**DOI:** 10.1155/2013/565271

**Published:** 2013-11-20

**Authors:** Seong-Ho Ok, Jeong Yeol Han, Hui-Jin Sung, Seong Min Yang, Jungchul Park, Seong-Chun Kwon, Mun-Jeoung Choi, Ju-Tae Sohn

**Affiliations:** ^1^Department of Anesthesiology and Pain Medicine, Gyeongsang National University School of Medicine, Jinju 660-772, Republic of Korea; ^2^Department of Anesthesiology and Pain Medicine, Gyeongsang National University Hospital, Jinju 660-702, Republic of Korea; ^3^Department of Physiology, Kwandong University College of Medicine, Gangneung 201-701, Republic of Korea; ^4^Department of Oral and Maxillofacial Surgery, Gyeongsang National University Hospital, Jinju 660-702, Republic of Korea; ^5^Department of Anesthesiology and Pain Medicine, Institute of Health Sciences, Gyeongsang National University School of Medicine, Gyeongsang National University Hospital, Jinju 660-772, Republic of Korea

## Abstract

This study investigated endothelium-derived vasodilators and potassium channels involved in the modulation of ropivacaine-induced contraction. In endothelium-intact rat aortae, ropivacaine concentration-response curves were generated in the presence or absence of the following inhibitors: the nonspecific nitric oxide synthase (NOS) inhibitor *N* ^**ω**^-nitro-L-arginine methyl ester (L-NAME), the neuronal NOS inhibitor *N* ^**ω**^-propyl-L-arginine hydrochloride, the inducible NOS inhibitor 1400W dihydrochloride, the nitric oxide-sensitive guanylyl cyclase (GC) inhibitor ODQ, the NOS and GC inhibitor methylene blue, the phosphoinositide-3 kinase inhibitor wortmannin, the cytochrome p450 epoxygenase inhibitor fluconazole, the voltage-dependent potassium channel inhibitor 4-aminopyridine (4-AP), the calcium-activated potassium channel inhibitor tetraethylammonium (TEA), the inward-rectifying potassium channel inhibitor barium chloride, and the ATP-sensitive potassium channel inhibitor glibenclamide. The effect of ropivacaine on endothelial nitric oxide synthase (eNOS) phosphorylation in human umbilical vein endothelial cells was examined by western blotting. Ropivacaine-induced contraction was weaker in endothelium-intact aortae than in endothelium-denuded aortae. L-NAME, ODQ, and methylene blue enhanced ropivacaine-induced contraction, whereas wortmannin, *N* ^**ω**^-propyl-L-arginine hydrochloride, 1400W dihydrochloride, and fluconazole had no effect. 4-AP and TEA enhanced ropivacaine-induced contraction; however, barium chloride and glibenclamide had no effect. eNOS phosphorylation was induced by ropivacaine. These results suggest that ropivacaine-induced contraction is attenuated primarily by both endothelial nitric oxide and voltage-dependent potassium channels.

## 1. Introduction

Ropivacaine is an aminoamide local anesthetic with a long duration that produces vasoconstriction both *in vivo* and *in vitro*, suggesting that intrinsic vasoconstriction induced by ropivacaine contributes to the drug's long-lasting analgesic effect [1–4]. Ropivacaine produces vasoconstriction at low concentrations, followed by vasodilation at 1 × 10^−3^ M [4]. The clinical profile of ropivacaine is similar to that of racemic bupivacaine, but its toxicity is relatively low compared with that of bupivacaine [5]. Ropivacaine is an aminoamide local anesthetic of the *n*-alkyl-substituted pipecolyl xylidine family, which includes levobupivacaine and mepivacaine [5]. Vasoconstriction induced by levobupivacaine and mepivacaine is attenuated by endothelial nitric oxide (NO) [6–8]. In endothelium-denuded aortae, ropivacaine-induced contraction is mediated mainly by the lipoxygenase pathway and partly by the cyclooxygenase pathway [4]. However, in endothelium-intact aortae, endothelium-derived vasodilators, including NO, endothelium-derived hyperpolarizing factor (EDHF), and prostacyclin, are involved in the modulation of vascular tone via vasodilation [9]. Ropivacaine induces endothelial NO-dependent relaxation in isolated vessels precontracted with phenylephrine and attenuates phenylephrine-induced contraction [10, 11]. In addition, the change of the membrane potential of vascular smooth muscle induced by the activation or inhibition of various potassium channels, including voltage-dependent, calcium-activated, inward-rectifying, and adenosine triphosphate-sensitive potassium channels, modulates vascular tone via vasodilation and vasoconstriction [12]. However, the endothelium-derived vasodilators and various potassium channels involved in the modulation of ropivacaine-induced contraction remain unknown. Therefore, the goal of this *in vitro* study was to investigate both endothelium-derived vasodilators and potassium channels primarily involved in modulating ropivacaine-induced contraction in isolated endothelium-intact aortae.

## 2. Materials and Methods

All experimental procedures and protocols were approved by the Institutional Animal Care and Use Committee (Jinju, Gyeongnam, Republic of Korea) at Gyeongsang National University and were performed in accordance with the Guide for the Care and Use of Laboratory Animals prepared by the National Academy of Sciences.

### 2.1. Preparation of Aortic Rings for Tension Measurement

Experimental preparation was performed as previously described [13]. Male Sprague-Dawley rats weighing 250–300 g were anesthetized via intramuscular injections of Zoletil 50 (15 mg/kg).  The descending thoracic aorta was dissected free, and surrounding connective tissues and fat were removed under microscopic guidance in a Krebs solution bath (118 mM NaCl, 4.7 mM KCl, 1.2 mM MgSO_4_, 1.2 mM KH_2_PO_4_, 2.4 mM CaCl_2_, 25 mM NaHCO_3_, and 11 mM glucose). The aorta was cut into 2.5 mm rings, suspended on Grass isometric transducers (FT-03, Grass Instrument, Quincy, MA, USA) under a 3.0 g resting tension in 10 mL of Krebs bath at 37°C, and aerated continuously with 95% O_2_ and 5% CO_2_ to maintain the pH within the range of 7.35–7.45. The rings were equilibrated for 120 min, changing the bathing solution every 30 min. Endothelium was removed from some aortic rings by inserting a 25-gauge needle tip into the lumen of the rings and gently rubbing for a few seconds. Once phenylephrine (1 × 10^−7^ M)-induced contraction had stabilized, acetylcholine (1 × 10^−5^ M) was added to assess the endothelial integrity. Endothelial integrity was confirmed by the observation of more than 70% acetylcholine-induced relaxation. Contraction in response to isotonic 60 mM KCl was measured for all aortic rings and defined as the reference value (100%). After washing out the KCl from the organ bath and allowing a return to the baseline resting tension, a cumulative concentration-response curve induced by ropivacaine was obtained as described in subsequent sections.

### 2.2. Experimental Protocols

The first series of experiment assessed the effect of endothelial denudation and nonspecific nitric oxide synthase (NOS) inhibitor *N* ^*ω*^-nitro-l-arginine methyl ester (l-NAME, 1 × 10^−4^ M) on the cumulative concentration (1 × 10^−5^ to 1 × 10^−3^ M)-response curves induced by ropivacaine in isolated aortae. l-NAME was directly added to the organ bath containing endothelium-intact aorta 20 min before the addition of ropivacaine. Subsequent concentrations of ropivacaine were directly added to the organ bath after the previous concentration had produced a sustained and stable response.

The second series of experiments assessed the cumulative concentration-response curves induced by ropivacaine in isolated endothelium-intact aortae in the presence or absence of the following inhibitors: the neuronal NOS inhibitor *N* ^*ω*^-propyl-l-arginine hydrochloride (5 × 10^−8^ M), the inducible NOS inhibitor 1400W dihydrochloride (1 × 10^−6^ M), the NO-sensitive guanylyl cyclase (GC) inhibitor 1H-[1,2, 4] oxadiazolo[4,3-*a*]quinoxalin-1-one (ODQ, 1 × 10^−6^ and 1 × 10^−5^ M), the NOS and GC inhibitor methylene blue (1 × 10^−6^ M), the cytochrome P450 epoxygenase inhibitor fluconazole (1 × 10^−5^ M), and the cyclooxygenase inhibitor indomethacin (1 × 10^−5^ and 3 × 10^−5^ M). The aforementioned inhibitors were directly added to the organ bath 20 min before the addition of ropivacaine. Inhibitor concentrations were chosen on the basis of the concentrations used in previous experiments similar to this experiment [6, 10, 13–18].

The third series of experiments assessed which specific potassium channels are primarily involved in the attenuation of ropivacaine-induced contraction in endothelium-intact aortae. In endothelium-intact aortae, ropivacaine concentration-response curves were generated in the presence or absence of the following potassium channel inhibitors: the voltage-dependent potassium channel inhibitor 4-aminopyridine (4-AP, 2 × 10^−3^ M), the calcium-activated potassium channel inhibitor tetraethylammonium (TEA, 2 × 10^−3^ M), the adenosine triphosphate-sensitive potassium channel inhibitor glibenclamide (1 × 10^−5^ M), and the inward-rectifying potassium channel inhibitor barium chloride (3 × 10^−5^ M) [19–22]. In addition, in the endothelium-intact aortae pretreated with 1 × 10^−4^ M l-NAME, cumulative ropivacaine concentration-response curves were generated in the presence or absence of either 4-AP (2 × 10^−3^ M) or TEA (2 × 10^−3^ M). In endothelium-intact aortae, cumulative phenylephrine concentration (1 × 10^−8^ to 1 × 10^−5^ M)-response curves were generated in the presence or absence of either 4-AP (2 × 10^−3^ M) or TEA (2 × 10^−3^ M). We also investigated whether ropivacaine-induced contraction involves endothelium-independent activation of voltage-dependent and calcium-activated potassium channels of vascular smooth muscle. After the ropivacaine (10^−4^ M)-induced contraction in endothelium-denuded aortae reached a plateau, TEA (2 × 10^−3^, 5 × 10^−3^, 1 × 10^−2^ M) or 4-AP (2 × 10^−3^, 5 × 10^−3^, 1 × 10^−2^ M) was cumulatively added to the organ bath to generate cumulative concentration-response curves for TEA or 4-AP. 

Finally, we assessed the ropivacaine concentration-response curves in endothelium-intact aortae in the presence or absence of the phosphoinositide-3 kinase (PI3K) inhibitor wortmannin (1 × 10^−7^ M) to determine whether the NO-mediated attenuation of ropivacaine-induced contraction is associated with the pathway involving PI3K-Akt-endothelial nitric oxide synthase (eNOS) [23, 24].

### 2.3. Cell Culture

Human umbilical vein endothelial cells (HUVECs; EA.hy 926 cells, American Type Culture Collection, Manassas, VA, USA) were grown in Dulbecco's modified Eagle's medium (DMEM), supplemented with 10% fetal bovine serum (FBS), 2 mmol/L l-glutamine, 100 IU/mL penicillin, and 10 *μ*g/mL streptomycin as previously described [6]. Cells were cultured in 100 mm dishes and grown in a humidified 5% CO_2_ incubator. HUVECs were plated at a density of 1 × 10^7^ cells per 100 mm dish. Cells were used between passage numbers 6 and 12.

### 2.4. Cell Stimulation

Cells were plated at a density of 1 × 10^7^ cells per 100 mm dish. The cells were stimulated with ropivacaine (1 × 10^−4^ M). To detect phosphorylated eNOS (p-eNOS), cells were treated with ropivacaine (1 × 10^−4^ M) for 5, 10, 30, and 60 min, harvested, and subjected to western blot analysis.

### 2.5. Western Blot Analysis

Western blot analysis was performed as previously described [6]. Briefly, cells were lysed in PRO-PREP protein extract solution to isolate total cell extracts. After centrifugation at 16,000 ×g for 20 min at 4°C, the protein concentration was determined by the Bradford method. Thirty micrograms of protein was subjected to 10% sodium dodecyl sulfate (SDS)-polyacrylamide gel electrophoresis. The separated proteins were transferred to a polyvinylidene difluoride membrane using the SD semidry transfer cell system (Bio-Rad, Hercules, CA, USA). The membranes were incubated with primary antibodies (anti-eNOS antibodies: rabbit polyclonal, Cell Signaling Technology, Beverly, MA, USA; anti-phospho-eNOS antibodies: Ser1777 rabbit polyclonal, Cell Signaling Technology) at a 1 : 500 concentration (4 *μ*g/mL) in 5% skim milk in Tris-buffered saline with Tween (TBST) overnight at 4°C, and the bound antibody was detected by horseradish peroxidase-conjugated anti-rabbit IgG. The membranes were washed and then developed using the Luminol Reagent system (Animal Genetics, Suwon, Republic of Korea).

### 2.6. Materials

All drugs were of the highest purity available commercially. Phenylephrine, l-NAME, 1400W dihydrochloride, ODQ, indomethacin, wortmannin, 4-AP, TEA, barium chloride, and glibenclamide were obtained from Sigma-Aldrich (Saint Louis, MO, USA). *N* ^*ω*^-propyl-l-arginine hydrochloride was obtained from Tocris Bioscience (Bristol, UK). Methylene blue and fluconazole were purchased from SALF Laboratorio Farmacologico (Bergamo, Italy) and Pfizer Global Manufacturing (France), respectively. Ropivacaine was donated by AstraZeneca Korea (Seoul, Republic of Korea). Zoletil 50 was purchased from Virbac (Virbac Laboratories, Carros, France). DMEM, FBS, penicillin, streptomycin, and glutamine were supplied by Gibco BRL (Rockville, MD, USA). All concentrations are expressed as the final molar concentration in the organ bath. ODQ, *N* ^*ω*^-propyl-l-arginine hydrochloride, 1400W dihydrochloride, wortmannin, indomethacin, and glibenclamide were dissolved in dimethyl sulfoxide (DMSO) (final organ bath concentration: 0.1% DMSO). Unless stated otherwise, all other drugs were dissolved in distilled water.

### 2.7. Data Analysis

Data are expressed as the mean ± SD. Contractile responses induced by ropivacaine are expressed as the percentage of the maximum contraction in response to isotonic 60 mM KCl. Vascular responses induced by TEA or 4-AP in endothelium-denuded aortae precontracted with 1 × 10^−4^ M ropivacaine are expressed as the percent change from baseline precontraction induced by 1 × 10^−4^ M ropivacaine. *N* indicates the number of rats from which descending thoracic aortic rings were derived. The effects of endothelial denudation and various inhibitors on the concentration-response curves induced by ropivacaine or phenylephrine were analyzed by two-way analysis of variance (ANOVA) followed by Bonferroni's post-hoc test using GraphPad Prism version 5.0 for Windows (GraphPad Software, San Diego, CA, USA). The band intensities from western blotting analysis were analyzed by Student's *t*-test. Reponses to each concentration of ropivacaine, 4-AP, and TEA were analyzed by repeated-measures ANOVA followed by Bonferroni's post-hoc test. *P* values less than 0.05 were considered significant.

## 3. Results

**Figure 1:**
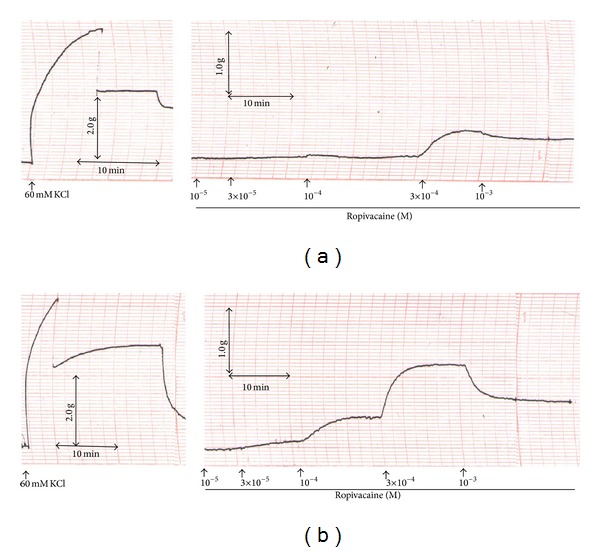
Traces showing the change in tension in endothelium-intact (a) and endothelium-denuded (b) aortae in response to 60 mM KCl and ropivacaine.

**Figure 2:**
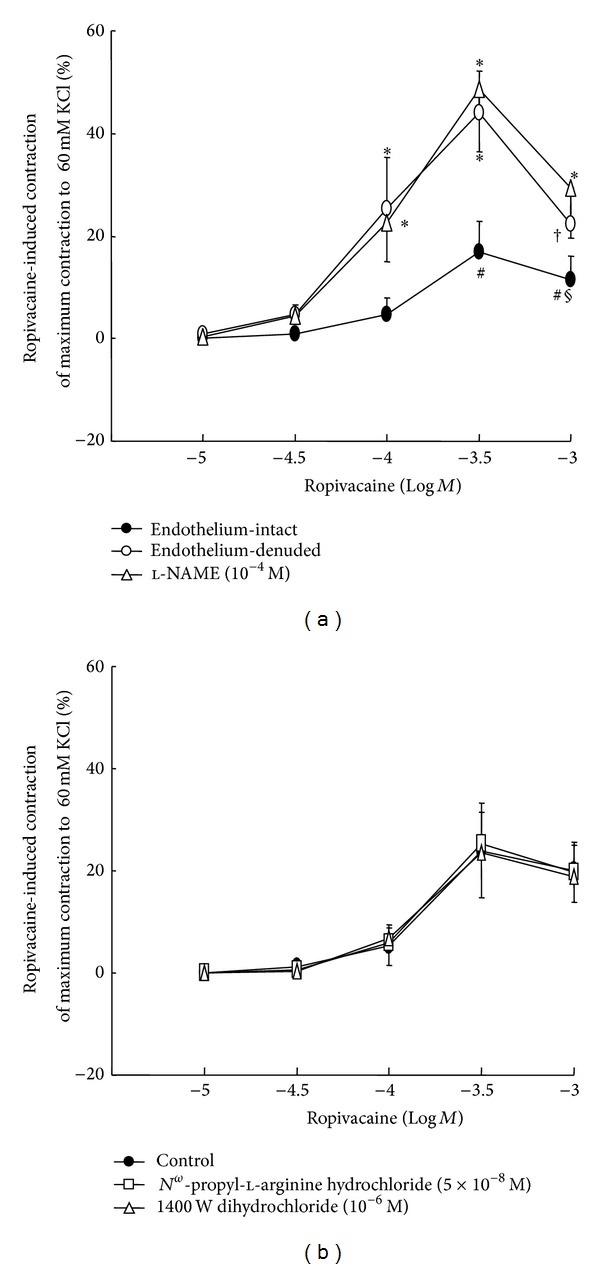
(a) The effect of endothelial denudation and *N* ^*ω*^-nitro-l-arginine methyl ester (l-NAME) on ropivacaine concentration-response curves in isolated aortae. Data are shown as the mean ± SD and expressed as a percentage of the maximal contraction induced by isotonic 60 mM KCl (100% = 2.29 ± 0.19 g [*n* = 7], 100% = 2.78 ± 0.39 g [*n* = 6], and 100% = 2.34 ± 0.33 g [*n* = 7] for untreated endothelium-intact aortae, untreated endothelium-denuded aortae, and endothelium-intact aortae treated with 1 × 10^−4^ M l-NAME, resp.). *N* indicates the number of rats from which descending thoracic aortic rings were derived. *P < 0.001 and ^†^ *P* < 0.05 versus endothelium-intact aortae. ^#^ *P* < 0.001 versus 1 × 10^−5^ M ropivacaine and ^§^ *P* < 0.05 versus 3 × 10^−4^ M in endothelium-intact aortae. (b) The effect of *N* ^*ω*^-propyl-l-arginine hydrochloride and 1400W dihydrochloride on ropivacaine concentration-response curves in endothelium-intact aortae. Data are shown as the mean ± SD and expressed as a percentage of the maximal contraction induced by isotonic 60 mM KCl (100% = 2.44 ± 0.46 g [*n* = 6], 100% = 2.28 ± 0.27 g [*n* = 6], and 100% = 2.33 ± 0.33 g [*n* = 6] for untreated endothelium-intact aortae, endothelium-intact aortae treated with 5 × 10^−8^ M *N* ^*ω*^-propyl-l-arginine hydrochloride, and endothelium-intact aortae treated with 1 × 10^−6^ M 1400W dihydrochloride, resp.). *N* indicates the number of rats from which descending thoracic aortic rings were derived.

Ropivacaine produced vasoconstriction at 3 × 10^−4^ M in endothelium-intact aortae, followed by vasodilation at 1 × 10^−3^ M (3 × 10^−4^ M: *P* < 0.001 versus 1 × 10^−5^ M; 1 × 10^−3^ M: *P* < 0.05 versus 3 × 10^−4^ M; Figures 1 and 2(a)).

**Figure 3:**
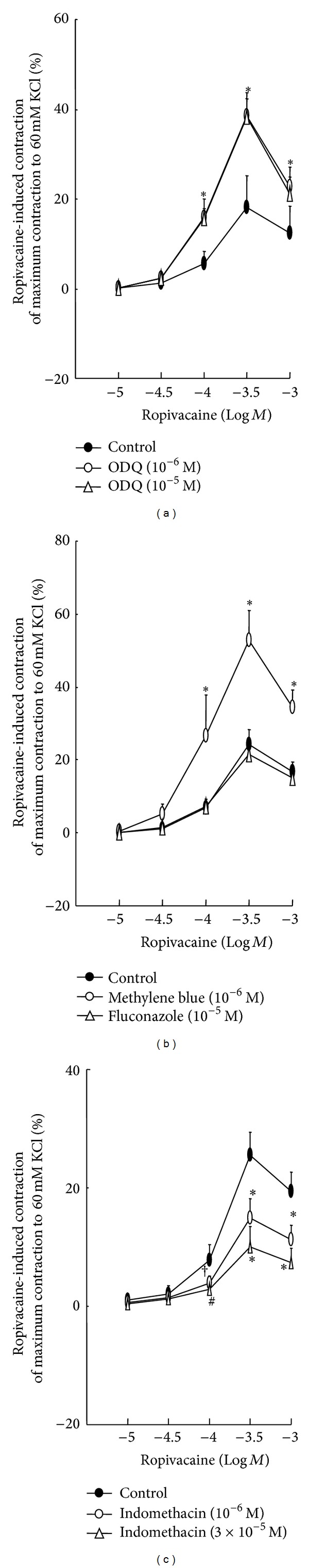
The effect of 1H-[1,2, 4]oxadiazolo[4,3-a]quinoxalin-1-one (ODQ) (a), methylene blue (b), fluconazole (b), and indomethacin (c) on ropivacaine concentration-response curves in endothelium-intact aortae. Data are shown as the mean ± SD and expressed as a percentage of the maximal contraction induced by isotonic 60 mM KCl. *N* indicates the number of rats from which descending thoracic aortic rings were derived. (a) 100% = 2.40 ± 0.48 g (*n* = 6), 100% = 2.55 ± 0.55 g (*n* = 6), and 100% = 2.70 ± 0.61 g (*n* = 6) for untreated endothelium-intact aortae, endothelium-intact aortae treated with 1 × 10^−6^ M ODQ, and endothelium-intact aortae treated with 1 × 10^−5^ M ODQ, respectively. (b) 100% = 2.00 ± 0.32 g (*n* = 9), 100% = 2.12 ± 0.40 g (*n* = 7), and 100% = 2.10 ± 0.42 g (*n* = 6) for untreated endothelium-intact aortae, endothelium-intact aortae treated with 1 × 10^−6^ methylene blue, and endothelium-intact aortae treated with 1 × 10^−5^ M fluconazole, respectively. (c) 100% = 2.08 ± 0.27 g (*n* = 6), 100% = 2.09 ± 0.40 g (*n* = 6), and 100% = 2.47 ± 0.67 g (*n* = 6) for untreated endothelium-intact aortae, endothelium-intact aortae treated with 1 × 10^−5^ M indomethacin, and endothelium-intact aortae treated with 3 × 10^−5^ M indomethacin, respectively. (a) and (b): *P < 0.001 versus control. (c): *P < 0.001, ^†^ *P* < 0.05, and ^#^ *P* < 0.01 versus control.

Ropivacaine-induced contraction was weaker in endothelium-intact aortae than in endothelium-denuded aortae (*P* < 0.05 versus endothelium-denuded aortae at 1 × 10^−4^ to 1 × 10^−3^ M ropivacaine; Figures 1 and 2(a)), suggesting that attenuation of ropivacaine-induced contraction is endothelium dependent. Pretreatment of endothelium-intact aortae with inhibitors including l-NAME (1 × 10^−4^ M), *N* ^*ω*^-propyl-l-arginine hydrochloride (5 × 10^−8^ M), 1400W dihydrochloride (1 × 10^−6^ M), ODQ (1 × 10^−5^ M), methylene blue (1 × 10^−6^ M), fluconazole (1 × 10^−5^ M), indomethacin (3 × 10^−5^ M), wortmannin (1 × 10^−7^ M), 4-AP (2 × 10^−3^ M), TEA (2 × 10^−3^ M), barium chloride (3 × 10^−5^ M), and glibenclamide (1 × 10^−5^ M) did not significantly alter the baseline resting tension (supplementary Figure 1 in Supplementary Material available online at http://dx.doi.org/10.1155/2013/565271). Pretreatment with the nonspecific NOS inhibitor l-NAME (1 × 10^−4^ M) significantly increased ropivacaine-induced contraction in endothelium-intact aortae (*P* < 0.001 versus endothelium-intact aortae at 1 × 10^−4^ to 1 × 10^−3^ M; Figure 2(a)), whereas the neuronal NOS inhibitor *N* ^*ω*^-propyl-l-arginine hydrochloride (5 × 10^−8^ M) and the inducible NOS inhibitor 1400W dihydrochloride (1 × 10^−6^ M) had no effect (Figure 2(b)), suggesting that endothelium-dependent attenuation of ropivacaine-induced contraction involves endothelial NO. Pretreatment with the NO-sensitive GC inhibitor ODQ (1 × 10^−6^ and 1 × 10^−5^ M) and the NOS and GC inhibitor methylene blue (1 × 10^−6^ M) significantly increased ropivacaine-induced contraction in endothelium-intact aortae (*P* < 0.001 versus control at 1 × 10^−4^ to 1 × 10^−3^ M; Figures 3(a) and 3(b)), suggesting that endothelium-dependent attenuation of ropivacaine-induced contraction involves the NO-GC pathway. The cytochrome P450 epoxygenase inhibitor fluconazole had no effect on ropivacaine-induced contraction in endothelium-intact aortae (Figure 3(b)), but the cyclooxygenase inhibitor indomethacin (1 × 10^−5^ and 3 × 10^−5^ M) attenuated ropivacaine-induced contraction (*P* < 0.05 versus control at 1 × 10^−4^ to 1 × 10^−3^ M; Figure 3(c)).

**Figure 4:**
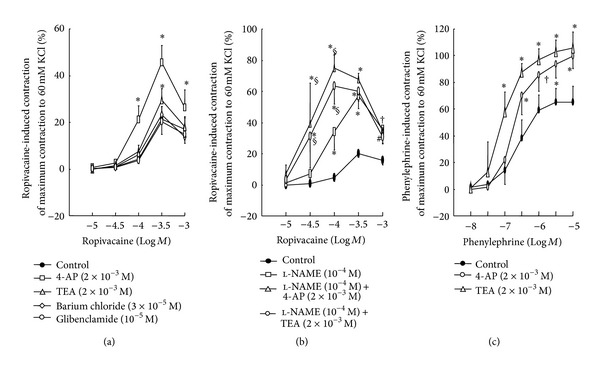
(a) and (b) The effect of 4-aminopyridine (4-AP), tetraethylammonium (TEA), barium chloride, and glibenclamide on ropivacaine concentration-response curves in endothelium-intact aortae without or with 1 × 10^−4^ M *N* ^*ω*^-nitro-l-arginine methyl ester (l-NAME). (c) The effect of 4-AP and TEA on phenylephrine concentration-response curves in endothelium-intact aortae. (a) and (b) Data are shown as the mean ± SD and expressed as a percentage of the maximal contraction induced by isotonic 60 mM KCl. *N* indicates the number of rats from which descending thoracic aortic rings were derived. (a) 100% = 2.38 ± 0.27 g (*n* = 10), 100% = 2.48 ± 0.47 g (*n* = 6), 100% = 2.20 ± 0.22 g (*n* = 6), 100% = 2.32 ± 0.31 g (*n* = 5), and 100% = 2.39 ± 0.31 g (*n* = 5) for untreated endothelium-intact aortae and endothelium-intact aortae treated with 2 × 10^−3^ M 4-AP, 2 × 10^−3^ M TEA, 3 × 10^−5^ M barium chloride, and 1 × 10^−5^ M glibenclamide, respectively. (b) 100% = 2.37 ± 0.22 g (*n* = 6), 100% = 2.81 ± 0.37 g (*n* = 6), 100% = 2.71 ± 0.30 g (*n* = 6), and 100% = 2.77 ± 0.14 g (*n* = 6) for untreated endothelium-intact aortae, endothelium-intact aortae pretreated with 1 × 10^−4^ M l-NAME alone, endothelium-intact aortae pretreated with 1 × 10^−4^ M l-NAME plus 2 × 10^−3^ M 4-AP, and endothelium-intact aortae pretreated with 1 × 10^−4^ M l-NAME plus 2 × 10^−3^ M TEA, respectively. (c) Data are shown as the mean ± SD and expressed as a percentage of the maximal contraction induced by isotonic 60 mM KCl (100% = 2.74 ± 0.32 g (*n* = 5), 100% = 2.77 ± 0.35 g (*n* = 5), and 100% = 2.66 ± 0.38 g (*n* = 5) for untreated endothelium-intact aortae and endothelium-intact aortae treated with 2 × 10^−3^ M 4-AP and 2 × 10^−3^ M TEA, resp.). *N* indicates the number of descending thoracic aortic rings. (a): *P < 0.001 versus control. (b) *P < 0.001, ^†^ *P* < 0.01 and ^#^ *P* < 0.05 versus control. ^§^ *P* < 0.001 versus 1 × 10^−4^ M l-NAME alone. (c) *P < 0.001 and ^†^ *P* < 0.01 versus control.

Pretreatment with the voltage-dependent potassium channel inhibitor 4-AP (2 × 10^−3^ M) greatly enhanced ropivacaine-induced contraction in endothelium-intact aortae (*P* < 0.001 versus control at 1 × 10^−4^ to 1 × 10^−3^ M), and pretreatment with the calcium-activated potassium channel inhibitor TEA (2 × 10^−3^ M) slightly increased ropivacaine-induced maximal contraction (*P* < 0.001 versus control at 3 × 10^−4^ M) (Figure 4(a)), suggesting that ropivacaine-induced contraction is attenuated by voltage-dependent and calcium-activated potassium channels. However, pretreatment with the inward-rectifying potassium channel inhibitor barium chloride (3 × 10^−5^ M) and the adenosine triphosphate-sensitive potassium channel inhibitor glibenclamide (1 × 10^−5^ M) had no effect on ropivacaine-induced contraction in endothelium-intact aortae (Figure 4(a)). Ropivacaine-induced contraction was stronger in endothelium-intact aortae pretreated with l-NAME (1 × 10^−4^ M) plus 4-AP (2 × 10^−3^ M) or l-NAME (1 × 10^−4^ M) plus TEA (2 × 10^−3^ M) than in endothelium-intact aortae pretreated with l-NAME (1 × 10^−4^ M) alone (*P* < 0.001 versus 1 × 10^−4^ M l-NAME alone at 3 × 10^−5^ and 1 × 10^−4^ M; Figure 4(b)). Pretreatment with 4-AP (2 × 10^−3^ M) or TEA (2 × 10^−3^ M) enhanced phenylephrine-induced contraction in endothelium-intact aortae (*P* < 0.01 versus control at 3 × 10^−7^ to 10^−5^ M; Figure 4(c)), suggesting that phenylephrine-induced contraction is attenuated by voltage-dependent and calcium-activated potassium channels.

**Figure 5:**
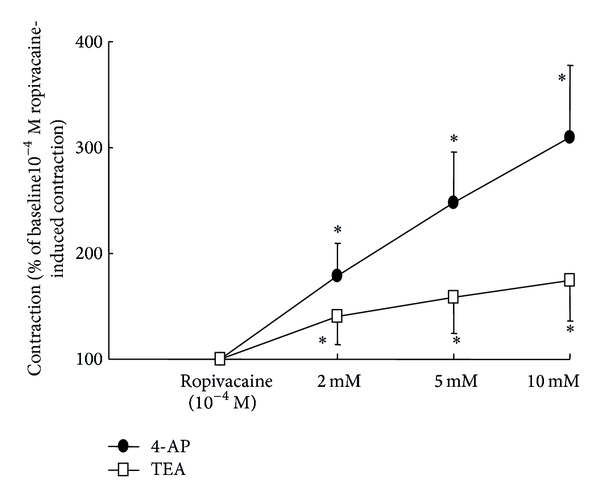
Cumulative concentration-response curves induced by 4-aminopyridine (4-AP) and tetraethylammonium (TEA) in endothelium-denuded aortae precontracted with 1 × 10^−4^ M ropivacaine. Data are shown as the mean ± SD and expressed as a percentage of the maximal contraction induced by ropivacaine (1 × 10^−4^ M) (100% = 0.94 ± 0.28 g (*n* = 10) and 100% = 0.97 ± 0.25 g (*n* = 10) for endothelium-denuded aortae with 4-AP and TEA, resp.). *N* indicates the number of descending thoracic aortic rings. *P < 0.001 versus ropivacaine (1 × 10^−4^ M).

4-AP (2 × 10^−3^ to 10^−2^ M) and TEA (2 × 10^−3^ to 10^−2^ M) induced contraction in endothelium-denuded aortae that were precontracted with ropivacaine (1 × 10^−4^ M) (Figure 5, *P* < 0.001), suggesting that ropivacaine-induced contraction involves endothelium-independent activation of voltage-dependent and calcium-activated potassium channels of vascular smooth muscle.

**Figure 6:**
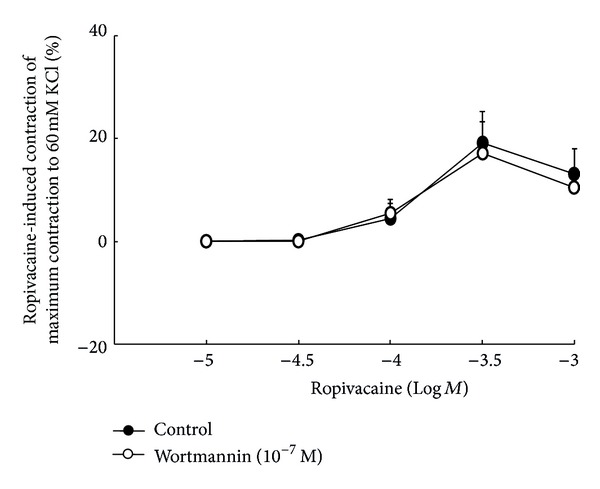
The effect of wortmannin (1 × 10^−7^ M) on ropivacaine concentration-response curves in endothelium-intact aortae. Data are shown as the mean ± SD and expressed as a percentage of the maximal contraction induced by isotonic 60 mM KCl (100% = 2.61 ± 0.14 g [*n* = 5] and 100% = 2.46 ± 0.20 g [*n* = 5] for untreated endothelium-intact aortae and endothelium-intact aortae treated with 1 × 10^−7^ M wortmannin, resp.). *N* indicates the number of rats from which descending thoracic aortic rings were derived.

The PI3K inhibitor wortmannin (1 × 10^−7^ M) had no effect on ropivacaine-induced contraction in endothelium-intact aortae (Figure 6), suggesting that endothelium-dependent attenuation of ropivacaine-induced contraction does not involve the PI3K-Akt-eNOS pathway.

**Figure 7:**
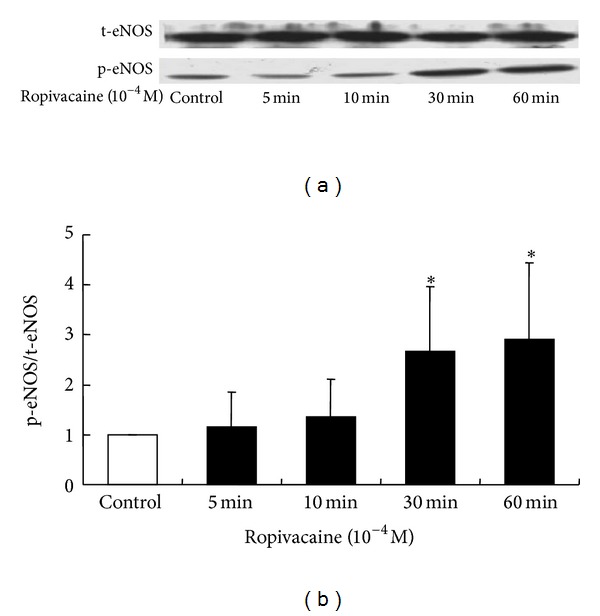
Effect of ropivacaine on the activation of endothelial nitric oxide synthase (eNOS; *n* = 4) by phosphorylation at Ser^1777^ in human umbilical vein endothelial cells (HUVECs). HUVECs were treated with ropivacaine (1 × 10^−4^ M) for 5, 10, 30, and 60 min. (a) Phosphorylation of eNOS was examined by western blotting, as described in Methods. (b) Band intensities at 5, 10, 30, and 60 min were assessed by scanning densitometry. Data are shown as the mean ± SD. *N* indicates the number of independent experiments. *P < 0.05 versus control. t-eNOS: total eNOS; P-eNOS: phosphorylated eNOS.

eNOS phosphorylation was induced in HUVECs at 30 and 60 min after treatment with 1 × 10^−4^ M ropivacaine (*P* < 0.05; Figure 7).

## 4. Discussion

This study presents novel information suggesting that ropivacaine-induced contraction is attenuated primarily by endothelial NO and voltage-dependent potassium channels in endothelium-intact aortae. The major findings of this *in vitro* study were as follows: (1) ropivacaine-induced contraction was attenuated in endothelium-intact aortae; (2) l-NAME, ODQ, and methylene blue enhanced ropivacaine-induced contraction in endothelium-intact aortae; (3) 4-AP and TEA enhanced ropivacaine-induced contraction in endothelium-intact aortae with or without l-NAME; (4) eNOS phosphorylation was induced by ropivacaine in HUVECs.

NO is produced from l-arginine in the endothelium by eNOS [9, 25]. Endothelial NO stimulates GC in the vascular smooth muscle and subsequently induces the formation of cyclic guanosine monophosphate (cGMP) and stimulation of cGMP-dependent protein kinase, which promote vascular smooth muscle relaxation [9, 25]. The attenuation of ropivacaine-induced contraction is endothelium dependent. In endothelium-intact aortae, the nonspecific NOS inhibitor l-NAME enhanced ropivacaine-induced contraction, whereas the highly selective neuronal NOS inhibitor *N* ^*ω*^-propyl-l-arginine hydrochloride and the inducible NOS inhibitor 1400W dihydrochloride did not affect contraction. Taken together, these results suggest that endothelium-dependent attenuation of ropivacaine-induced contraction is associated with eNOS. Ropivacaine produces endothelium-dependent vasodilation in isolated guinea pig aortae precontracted with phenylephrine via a pathway involving NO-GC [10]. In addition, ropivacaine attenuates phenylephrine-induced contraction of endothelium-intact aortae in an endothelial NO-dependent manner [11]. Similar to the results of previous studies that used different methods from those used here, our findings that the NOS inhibitor l-NAME, the NO-sensitive GC inhibitor ODQ, and the combined NOS and GC inhibitor methylene blue enhanced ropivacaine-induced contraction in endothelium-intact aortae suggest that endothelium-dependent attenuation of ropivacaine-induced contraction is associated with activation of the NO-GC-cGMP pathway [10, 11]. Ropivacaine-induced contraction is dependent on calcium influx via voltage-operated calcium channels [4, 26]. Ropivacaine-induced contraction appears to be mediated by cytosolic phospholipase A_2_ activated by calcium influx [4]. This calcium influx may contribute to activation of eNOS because the eNOS that produces NO binds calmodulin in a calcium-dependent manner [9]. PI3K stimulates Akt (protein kinase B) as a downstream signal molecule and subsequently induces eNOS phosphorylation and vasodilatation, which is a calcium-independent novel mechanism for eNOS activation [23]. The PI3K inhibitor wortmannin had no effect on ropivacaine-induced contraction (Figure 6), suggesting that endothelial NO-mediated attenuation of ropivacaine-induced contraction is not associated with the pathway involving PI3K-Akt-eNOS. Further research on the effect of ropivacaine on the endothelial intracellular concentration of free calcium, which is required for the classic signal pathway of eNOS activation, is needed to elucidate the detailed cellular mechanism of ropivacaine-induced NO release.

Reinforced by the results obtained from isometric tension measurements in the current study, ropivacaine induced eNOS phosphorylation in HUVECs. Because we used HUVECs instead of rat aortic endothelial cells, and considering the heterogeneity of endothelial cells, we should be very cautious about interpreting data obtained from western blotting using HUVECs [27]. In this *in vitro* study, the time (30 min) required for ropivacaine-induced eNOS phosphorylation in HUVECs appears to be slightly longer than that required for ropivacaine-induced contraction inhibited by endothelial NO release. This difference may be ascribed to differences in vessel location and species. In addition, both levobupivacaine and mepivacaine induce endothelium-dependent NO-mediated attenuation of vasoconstriction and eNOS phosphorylation [6–8, 16]. As ropivacaine belongs to the family of *n*-alkyl-substituted pipecolyl xylidine aminoamide local anesthetics that includes levobupivacaine and mepivacaine, endothelium-dependent NO-mediated attenuation of ropivacaine-induced contraction may be a common characteristic of this family of local anesthetics.

The activation of various potassium channels results in potassium efflux via the opening of potassium channels and subsequently induces membrane hyperpolarization, which leads to the relaxation of vascular smooth muscle through the inhibition of voltage-operated calcium channels [12]. In endothelium-intact aortae, ropivacaine-induced contraction was greatly enhanced by 4-AP and slightly enhanced by TEA, suggesting that ropivacaine-induced contraction involves both the primary activation of voltage-dependent potassium channels and the partial activation of calcium-activated potassium channels. Ropivacaine increases the intracellular free calcium concentration in vascular smooth muscle, which may contribute to the stimulation of calcium-activated potassium channels observed in this study [26]. Glibenclamide and barium chloride had no effect on ropivacaine-induced contraction in endothelium-intact aortae, suggesting that ropivacaine-induced contraction does not involve the activation of adenosine triphosphate-sensitive and inward-rectifying potassium channels. Procaine, an aminoamide local anesthetic, produces vasodilation in aortae precontracted with phenylephrine via both endothelial NO and endothelium-independent calcium-activated potassium channels [28]. Conversely, endothelial NO produced by endothelium-dependent vasodilators stimulates the opening of various potassium channels including voltage-dependent, calcium-activated, and adenosine triphosphate-sensitive potassium channels via the stimulation of cGMP-dependent protein kinase and subsequently produces vasodilation [20, 29, 30]. In the current study, the endothelium-dependent attenuation of ropivacaine-induced contraction appeared to involve endothelial NO release. If l-NAME-mediated enhancement of ropivacaine-induced contraction involves inactivation of the opening of potassium channels induced by a NO-mediated pathway, there would be no significant difference in ropivacaine-induced contraction between endothelium-intact aortae pretreated with l-NAME alone and endothelium-intact aortae pretreated with l-NAME plus potassium channel inhibitor (2 × 10^−3^ M TEA or 4-AP). However, as 4-AP- and TEA-mediated enhancement of ropivacaine-induced contraction was observed in l-NAME-pretreated endothelium-intact aortae (Figure 4(b)), these results suggest that ropivacaine-induced, voltage-dependent, and calcium-activated potassium channel activation may be mediated by an endothelial NO-independent mechanism. In addition, 4-AP or TEA produced vasoconstriction in endothelium-denuded aortae precontracted with ropivacaine (Figure 5). Taken together, these results suggest that ropivacaine-induced contraction is attenuated by two independent mechanisms including endothelial NO and endothelium-independent activation of voltage-dependent and calcium-activated potassium channels in vascular smooth muscle. Further research into the effect of potassium channel inhibitors on the voltage-dependent and calcium-activated potassium channel current induced by ropivacaine in vascular smooth muscle cells is needed to elucidate the detailed cellular mechanism. Local anesthetics including bupivacaine and ropivacaine inhibit voltage-dependent and tandem pore domain potassium channels, which may contribute to local anesthetic toxicity, whereas the activation of voltage-dependent and calcium-activated potassium channels accompanied by ropivacaine-induced vasoconstriction observed in this study may be associated with a negative feedback mechanism in which voltage-operated calcium channel-mediated vasoconstriction induced by a contractile agonist (e.g., phenylephrine) limits muscle contraction via the opening of voltage-dependent and calcium-activated potassium channels [12, 31, 32]. Furthermore, 4-AP and TEA increased phenylephrine-induced contraction in the present study (Figure 4(c)), suggesting that phenylephrine-induced contraction also induces the activation of voltage-dependent and calcium-activated potassium channels. Thus, the opening of voltage-dependent and calcium-activated potassium channels accompanied by ropivacaine-induced vasoconstriction appears to be associated with a nonspecific negative feedback mechanism that limits ropivacaine-induced vasoconstriction in vascular smooth muscle cells.

One of the major proposed mechanisms responsible for EDHF-induced vasodilation is potassium channel activation induced by epoxyeicosatrienoic acid, which is produced from arachidonic acid via cytochrome P450 epoxygenase [33]. The cytochrome P450 epoxygenase inhibitor fluconazole had no effect on ropivacaine-induced contraction, suggesting that cytochrome P450 epoxygenase-mediated EDHF-induced vasodilation does not contribute to the endothelium-dependent attenuation of ropivacaine-induced contraction. Indomethacin attenuated ropivacaine-induced contraction, suggesting that the endothelium-dependent attenuation of ropivacaine-induced contraction does not involve endothelial prostacyclin. Further investigation into the effect of ropivacaine on the production of arachidonic acid metabolite in endothelial cells is needed.

Ropivacaine at lower concentrations induces both vasoconstriction and decreased skin blood flow [1–4]. The combined topical application of ropivacaine and epinephrine does not further reduce sciatic nerve blood flow compared with the topical application of ropivacaine alone, suggesting that adding epinephrine to ropivacaine does not synergistically induce vasoconstriction, which may be due to the strong intrinsic vasoconstriction induced by ropivacaine alone [34]. The clinical relevance of ropivacaine-induced vasoconstriction revealed in this study must be tempered by the fact that the aorta is a conduit vessel, whereas blood flow is controlled by small-resistance arterioles such as rat mesenteric arteries with diameters of less than 100–300 *μ*m [35]. Even with this limitation, vasoconstriction induced by 3 × 10^−4^ M ropivacaine, which corresponds to 0.093% ropivacaine and is within the clinically relevant concentration (0.2%) of ropivacaine used for local infiltration, may contribute to the vasoconstriction and decreased blood flow observed in previous studies [1–4, 34]. As ropivacaine-induced contraction is attenuated by both endothelial NO and voltage-dependent and calcium-activated potassium channels, the magnitude of ropivacaine-induced contraction may be enhanced in patients with decreased endothelial function and impaired potassium channel function associated with hypertension and diabetes, leading to a longer duration of ropivacaine-induced analgesia [20].

In conclusion, these results suggest that ropivacaine-induced contraction is attenuated primarily by both endothelial NO release and the activation of voltage-dependent potassium channels. The activation of voltage-dependent and calcium-activated potassium channels that is induced by ropivacaine-induced contraction seems to be associated with a negative feedback mechanism. In addition, the endothelial NO-mediated attenuation of ropivacaine-induced contraction does not appear to involve the activation of the pathway associated with PI3K-Akt-eNOS.

## Supplementary Material

Traces showing the change in baseline resting tension in endothelium-intact aortae in response to various inhibitors

## References

[1] Cederholm I, Evers H, Löfström JB (1991). Effect of intradermal injection of saline or a local anaesthetic agent on skin blood flow—a methodological study in man. *Acta Anaesthesiologica Scandinavica*.

[2] Cederholm I, Evers H, Löfström JB (1992). Skin blood flow after intradermal injection of ropivacaine in various concentrations with and without epinephrine evaluated by laser doppler flowmetry. *Regional Anesthesia*.

[3] Nakamura K, Toda H, Kakuyama M (1993). Direct vascular effect of ropivacaine in femoral artery and vein of the dog. *Acta Anaesthesiologica Scandinavica*.

[4] Sung H-J, Sohn J-T, Park J-Y, Hwang EM, Baik JS, Ogawa K (2009). Direct effect of ropivacaine involves lipoxygenase pathway activation in rat aortic smooth muscle. *Canadian Journal of Anesthesia*.

[5] Casati A, Putzu M (2005). Bupivacaine, levobupivacaine and ropivacaine: are they clinically different?. *Best Practice and Research: Clinical Anaesthesiology*.

[6] Sung HJ, Choi MJ, Ok SH (2012). Mepivacaine-induced contraction is attenuated by endothelial nitric oxide release in isolated rat aorta. *Canadian Journal of Physiology and Pharmacology*.

[7] Baik JS, Sohn J-T, Ok S-H (2011). Levobupivacaine-induced contraction of isolated rat aorta is calcium dependent. *Canadian Journal of Physiology and Pharmacology*.

[8] Ok S-H, Sohn J-T, Baik J-S (2011). Lipid emulsion reverses levobupivacaine-induced responses in isolated rat aortic vessels. *Anesthesiology*.

[9] Busse R, Fleming I, Hecker M (1993). Signal transduction in endothelium-dependent vasodilatation. *European Heart Journal*.

[10] Lin PL, Huang HH, Fan SZ, Tsai MC, Lin CH, Huang CH (2007). Effect of ropivacaine on endothelium-dependent phenylephrine-induced contraction in guinea pig aorta. *Acta Anaesthesiologica Scandinavica*.

[11] Lee JU, Shin YS, Kim YH, Kim EJ, Kim HS (2004). Effect of ropivacaine on endothelium and nitric oxide in rat thoracic aortic rings. *Korean Journal of Anesthesiology*.

[12] Thorneloe KS, Nelson MT (2005). Ion channels in smooth muscle: regulators of intracellular calcium and contractility. *Canadian Journal of Physiology and Pharmacology*.

[13] Ok SH, Bae SI, Shim HS, Sohn JT (2012). Dexmedetomidine-induced contraction of isolated rat aorta is dependent on extracellular calcium concentration. *Korean Journal of Anesthesiology*.

[14] Kunze KL, Wienkers LC, Thummel KE, Trager WF (1996). Warfarin-Fluconazole I—inhibition of the human cytochrome P450-dependent metabolism of warfarin by fluconazole: in vitro studies. *Drug Metabolism and Disposition*.

[15] Stawicki SP, Sims C, Sarani B, Grossman MD, Gracias VH (2008). Methylene blue and vasoplegia: who, when, and how?. *Mini-Reviews in Medicinal Chemistry*.

[16] Choi YS, Jeong YS, Ok S-H (2010). The direct effect of levobupivacaine in isolated rat aorta involves lipoxygenase pathway activation and endothelial nitric oxide release. *Anesthesia and Analgesia*.

[17] El-Awady MSH, Smirnov SV, Watson ML (2008). Desensitization of the soluble guanylyl cyclase/cGMP pathway by lipopolysaccharide in rat isolated pulmonary artery but not aorta. *British Journal of Pharmacology*.

[18] Zhang HQ, Fast W, Marletta MA, Martasek P, Silverman RB (1997). Potent and selective inhibition of neuronal nitric oxide synthase by *N*
^*ω*^-propyl-L-arginine. *Journal of Medicinal Chemistry*.

[19] Kaya T, Gursoy S, Karadas B, Sarac B, Kafali H, Soydan AS (2003). High-concentration tramadol-induced vasodilation in rabbit aorta is mediated by both endothelium-dependent and -independent mechanisms. *Acta Pharmacologica Sinica*.

[20] Ko EA, Han J, Jung ID, Park WS (2008). Physiological roles of K^+^ channels in vascular smooth muscle cells. *Journal of Smooth Muscle Research*.

[21] Subramaniam G, Achike FI, Mustafa MR (2009). Effect of acidosis on the mechanism(s) of insulin-induced vasorelaxation in normal Wistar-Kyoto (WKY) rat aorta. *Regulatory Peptides*.

[22] Xue Y-L, Shi H-X, Murad F, Bian K (2011). Vasodilatory effects of cinnamaldehyde and its mechanism of action in the rat aorta. *Vascular Health and Risk Management*.

[23] Dimmeler S, Fleming I, Fisslthaler B, Hermann C, Busse R, Zeiher AM (1999). Activation of nitric oxide synthase in endothelial cells by Akt- dependent phosphorylation. *Nature*.

[24] Jin SN, Wen JF, Wang TT, Kang DG, Lee HS, Cho KW (2012). Vasodilatory effects of ethanol extract of Radix Paeoniae Rubra and its mechanism of action in the rat aorta. *Journal of Ethnopharmacology*.

[25] Sohn J-T, Kim H-J, Cho H-C, Shin I-W, Lee H-K, Chung Y-K (2004). Effect of etomidate on endothelium-dependent relaxation induced by acetylcholine in rat aorta. *Anaesthesia and Intensive Care*.

[26] Tokinaga Y, Ogawa K, Yu J, Kuriyama T, Minonishi T, Hatano Y (2007). Mechanism of the ropivacaine-induced increase in intracellular Ca^2+^ concentration in rat aortic smooth muscle. *Acta Anaesthesiologica Scandinavica*.

[27] Cines DB, Pollak ES, Buck CA (1998). Endothelial cells in physiology and in the pathophysiology of vascular disorders. *Blood*.

[28] Huang Y, Lau CW, Chan FL, Yao XQ (1999). Contribution of nitric oxide and K^+^ channel activation to vasorelaxation of isolated rat aorta induced by procaine. *European Journal of Pharmacology*.

[29] Han W-Q, Wong WT, Tian XY (2010). Contributory role of endothelium and voltage-gated potassium channels in apocynin-induced vasorelaxations. *Journal of Hypertension*.

[30] Gong L, Peng J, Fang L (2012). The vasorelaxant mechanisms of a Rho kinase inhibitor DL0805 in rat thoracic aorta. *Molecules*.

[31] Kindler CH, Yost CS, Gray AT (1999). Local anesthetic inhibition of baseline potassium channels with two pore domains in tandem. *Anesthesiology*.

[32] González T, Longobardo M, Caballero R, Delpón E, Tamargo J, Valenzuela C (2001). Effect of bupivacaine and a novel local anesthetic IQB-9302, on human cardiac K^+^ channels. *Journal of Pharmacology and Experimental Therapeutics*.

[33] Bryan RM, You J, Golding EM, Marrelli SP (2005). Endothelium-derived hyperpolarizing factor: a cousin to nitric oxide and prostacyclin. *Anesthesiology*.

[34] Bouaziz H, Iohom G, Estèbe J-P, Campana WM, Myers RR (2005). Effects of levobupivacaine and ropivacaine on rat sciatic nerve blood flow. *British Journal of Anaesthesia*.

[35] Christensen KL, Mulvany MJ (2001). Location of resistance arteries. *Journal of Vascular Research*.

