# Supplementary figures and images for: Ropivacaine-Induced Contraction Is Attenuated by Both Endothelial Nitric Oxide and Voltage-Dependent Potassium Channels in Isolated Rat Aortae
Source: Biomed Res Int. 2013 Nov 20;2013:565271. doi: 10.1155/2013/565271 (PMC3853310; doi:10.1155/2013/565271)

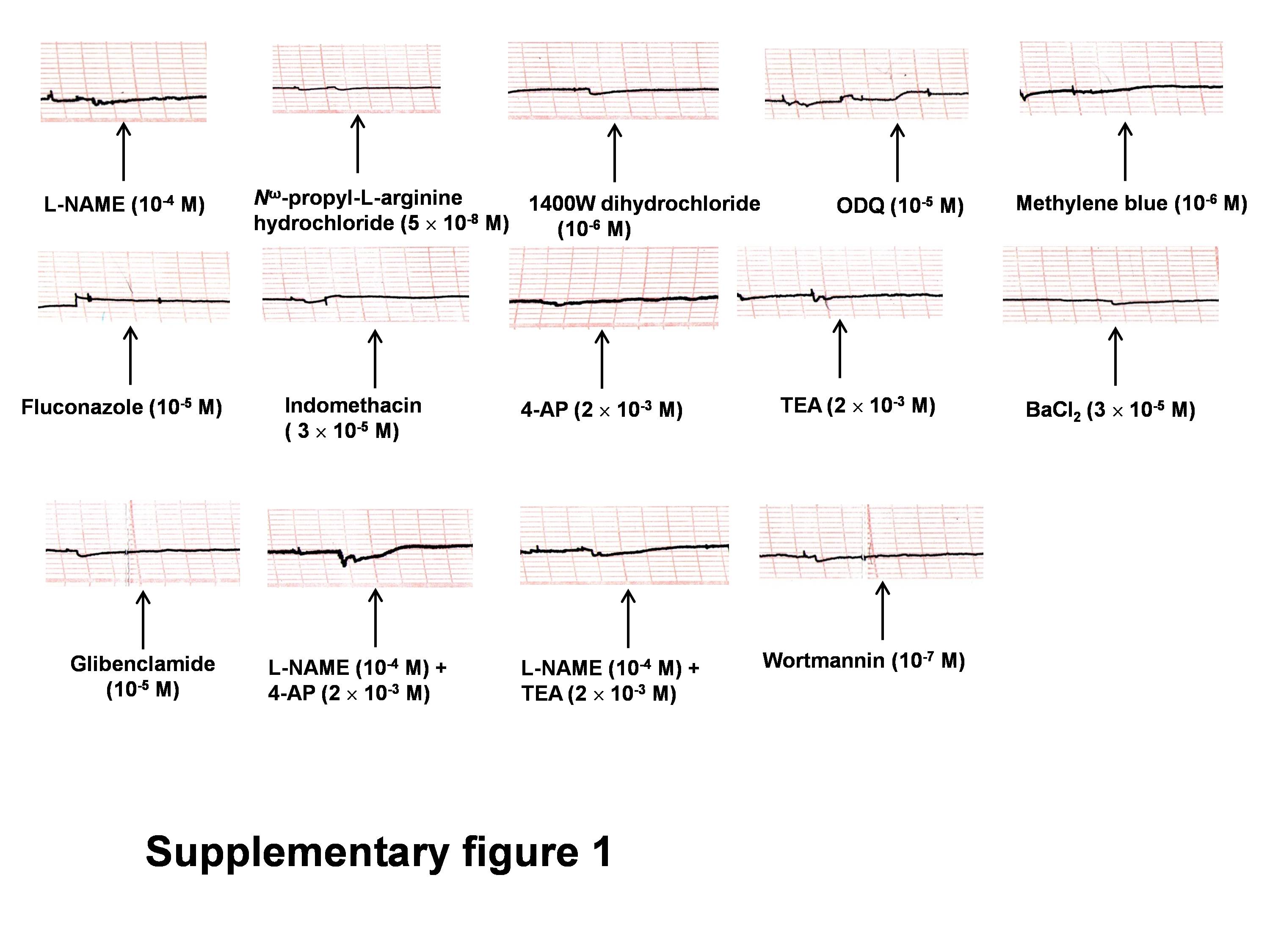

Supplement: Supplementary file 1 — Traces showing the change in baseline resting tension in endothelium-intact aortae in response to various inhibitors [file 565271.f1.jpg]
